# TTK inhibition increases cisplatin sensitivity in high-grade serous ovarian carcinoma through the mTOR/autophagy pathway

**DOI:** 10.1038/s41419-021-04429-6

**Published:** 2021-12-07

**Authors:** Gonghua Qi, Hanlin Ma, Yingwei Li, Jiali Peng, Jingying Chen, Beihua Kong

**Affiliations:** 1 Department of Obstetrics and Gynecology, Qilu Hospital, Shandong University, 250012 Jinan, China; 2 Gynecologic Oncology Key Laboratory of Shandong Province, Qilu Hospital, Shandong University, 250012 Jinan, China

**Keywords:** Chemotherapy, Targeted therapies, Autophagy, Diagnostic markers

## Abstract

High-grade serous ovarian cancer (HGSOC) is the most lethal gynecological malignancy. However, the molecular mechanisms underlying HGSOC development, progression, chemotherapy insensitivity and resistance remain unclear. Two independent GEO datasets, including the gene expression profile of primary ovarian carcinoma and normal controls, were analyzed to identify genes related to HGSOC development and progression. A KEGG pathway analysis of the differentially expressed genes (DEGs) revealed that the cell cycle pathway was the most enriched pathway, among which TTK protein kinase (TTK) was the only gene with a clinical-grade inhibitor that has been investigated in a clinical trial but had not been studied in HGSOC. TTK was also upregulated in cisplatin-resistant ovarian cancer cells from two other datasets. TTK is a regulator of spindle assembly checkpoint signaling, playing an important role in cell cycle control and tumorigenesis in various cancers. However, the function and regulatory mechanism of TTK in HGSOC remain to be determined. In this study, we observed TTK upregulation in patients with HGSOC. High TTK expression was related to a poor prognosis. Genetic and pharmacological inhibition of TTK impeded the proliferation of ovarian cancer cells by disturbing cell cycle progression and increasing apoptosis. TTK silencing increased cisplatin sensitivity by activating the mammalian target of rapamycin (mTOR) complex to further suppress cisplatin-induced autophagy in vitro. In addition, the enhanced sensitivity was partially diminished by rapamycin-mediated inhibition of mTOR in TTK knockdown cells. Furthermore, TTK knockdown increased the toxicity of cisplatin in vivo by decreasing autophagy. These findings suggest that the administration of TTK inhibitors in combination with cisplatin may lead to improved response rates to cisplatin in patients with HGSOC presenting high TTK expression. In summary, our study may provide a theoretical foundation for using the combination therapy of cisplatin and TTK inhibitors as a treatment for HGSOC in the future.

## Introduction

Epithelial ovarian cancer is the most lethal gynecological malignancy, and high-grade serous ovarian carcinoma (HGSOC) accounts for 70% of ovarian cancer cases and is the most common subtype with the highest mortality rate [1]. The 5-year overall survival rate of patients with HGSOC is approximately 30% [2, 3]. At present, the standard therapy for primary HGSOC is still surgery, followed by platinum-based combination chemotherapy, which causes cancer cell death by inducing DNA damage and cellular apoptosis [4, 5]. However, most relapses occur because ovarian cancer is insensitive or resistant to primary chemotherapy [2, 3]. However, the molecular mechanisms underlying HGSOC chemotherapy insensitivity and resistance remain unclear.

TTK protein kinase (TTK), also named monopolar spindle 1 (Mps1), is a dual specificity serine/threonine kinase and plays an important role in regulating spindle assembly checkpoint (SAC) signaling [6–8]. SAC, a surveillance mechanism in mitosis, guarantees the fidelity of chromosome segregation to maintain genome stability [9]. Thus, TTK plays vital roles in promoting the formation of the mitotic checkpoint complex, facilitating proper chromosome alignment, regulating cytokinesis and responding to DNA damage [10].

In addition, recent studies have revealed that TTK functions as an oncogene in a variety of cancers, such as glioblastoma, breast, liver, prostate, lung, bladder, gastric, colon and pancreatic cancer [11–22], and knocking down TTK expression inhibits tumor growth. Several small-molecule inhibitors targeting TTK have been developed and characterized in vitro and in vivo [23–36]. Meanwhile, some of the inhibitors have been tested in clinical trials, such as BAY1161909 (NCT02138812), BAY1217389 (NCT02366949), CFI-402257 (NCT03568422), and BOS172722 (NCT03328494) [37–40]. However, the function of TTK in ovarian cancer progression and the role of TTK inhibitors in ovarian cancer therapy have not been determined.

Inhibition of TTK enhances the efficacy of docetaxel in a murine triple-negative breast cancer model [33] and promotes the radiosensitivity of glioblastoma [11]. Furthermore, recent studies reported that genetic and pharmacological inhibition of TTK enhances radiosensitivity in basal-like breast cancer [15] and liver cancer [16] by inducing persistent DNA damage. Based on these findings, we speculated that TTK inhibition may increase cisplatin cytotoxicity and sensitivity in HGSOC.

## Results

### TTK is a key gene regulating the cell cycle pathway that contributes to the oncogenesis and chemoresistance of HGSOC

**Fig. 1:**
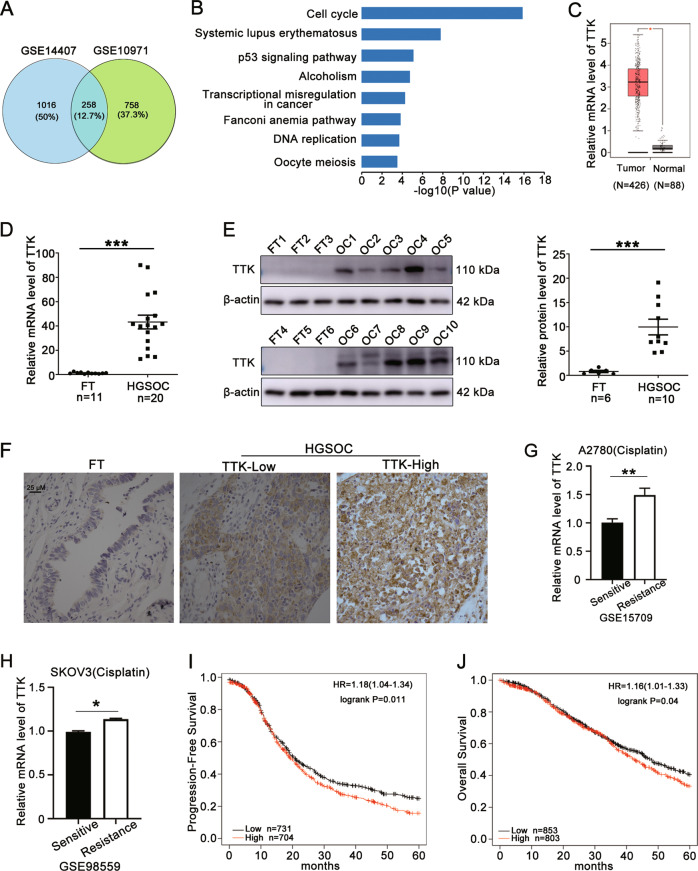
TTK is a key gene regulating the cell cycle pathway that contributes to the oncogenesis and chemoresistance of HGSOC. **A** Two GEO datasets (GSE14407 and GSE10971) were used to distinguish genes related to the oncogenesis of serous ovarian carcinoma (log2 FC > 1; *P* < 0.001). **B** The KEGG pathway analysis of overlapping genes in (**A**). **C** The mRNA expression of TTK in ovarian cancer (*n* = 426) and normal control tissues (*n* = 88) from the GEPIA database. **D** RT–qPCR analysis showing the TTK mRNA level in HGSOC and the comparison to FT tissues (FT, *n* = 11; HGSOC, *n* = 20). **E** The level of the TTK protein in HGSOC and FT tissues was detected using western blot assays (FT, FT1-FT6; HGSOC, OC1-OC10). **F** Representative IHC images of TTK staining in HGSOC and FT tissues (×400), scale bar: 25 µm. **G**, **H** The TTK mRNA level in cisplatin-resistant A2780 (GSE15709) and SKOV3 (GSE98559) cells. **I**, **J** PFS (**I**) and OS (**J**) analyses using the K−M plotter database based on TTK expression (data are mean ± SEM, *P < 0.05, **P < 0.01, ***P < 0.001, *n* = 3).

We identified genes involved in the tumorigenesis and chemoresistance of ovarian cancer by searching two independent datasets in the GEO database containing gene expression profiles for primary serous ovarian carcinoma and normal controls. A total of 258 overlapping upregulated differentially expressed genes (DEGs) were identified based on the criteria of log2(fold change) > 1 and *P* value < 0.001 (Fig. 1A). The KEGG pathway analysis of overlapping upregulated DEGs revealed that the cell cycle pathway was the most enriched pathway (Fig. 1B). Twenty-two DEGs participating in the cell cycle pathway were identified, and TTK was the only DEG with a clinical-grade inhibitor investigated in a clinical trial that had not been studied in HGSOC (Supplementary Table S1). Therefore, the role of TTK in the development of ovarian cancer was further investigated in our study. TTK was expressed at higher levels in ovarian cancer (Fig. 1C and Supplementary Fig. S1A–C) and other cancers (Supplementary Fig. S1D) than in the corresponding normal controls in several open access databases. Meanwhile, the TTK mRNA and protein levels detected in our patient samples were consistent with those in the online databases (Fig. 1D–F). In addition, the TTK expression level was increased in cisplatin-resistant A2780 and SKOV3 cells (Fig. 1G, H). Finally, progression-free survival (PFS) and overall survival (OS) analyses with Kaplan–Meier plotter showed that patients with high TTK expression had a poor prognosis (Fig. 1I, J). Collectively, TTK is a key cell cycle regulator that contributes to the oncogenesis, chemoresistance and poor prognosis of HGSOC. Thus, drugs targeting TTK might be a novel targeted therapy for patients with HGSOC.

### TTK depletion inhibits ovarian cancer cell proliferation by disturbing cell cycle progression

**Fig. 2:**
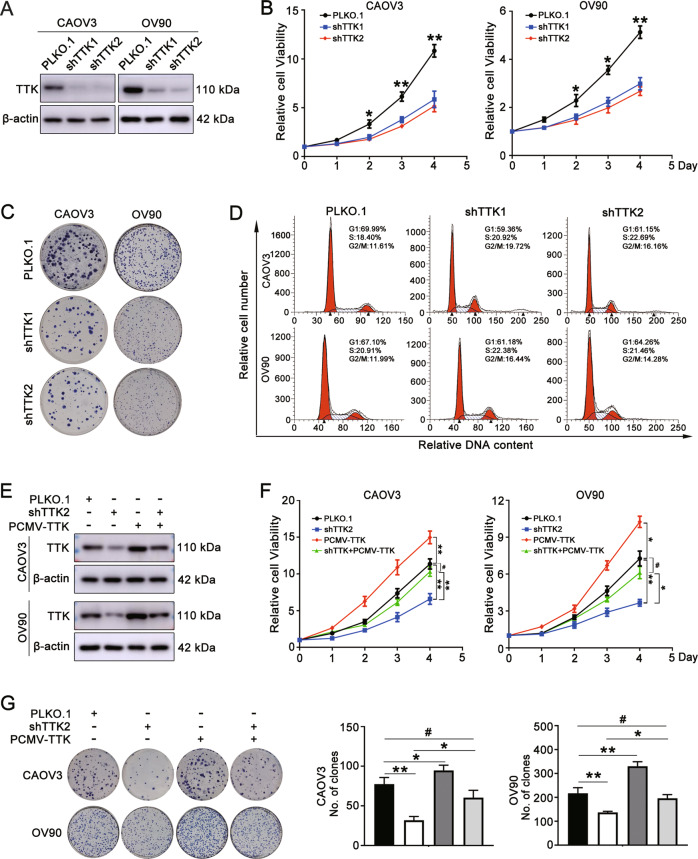
TTK depletion inhibits ovarian cancer cell proliferation by disturbing cell cycle progression. CAOV3 and OV90 cells were stably transfected with PLKO.1 or the TTK shRNA (shTTK1 and shTTK2) **A** The TTK protein level in CAOV3 and OV90 cells after TTK knockdown. **B** MTT assays showed the effect of TTK knockdown on the proliferation of CAOV3 and OV90 cells. **C** The effect of TTK inhibition on the colony formation ability of CAOV3 and OV90 cells. **D** Cell cycle analysis of CAOV3 and OV90 cells after TTK knockdown. The TTK overexpression plasmid (PCMV-TTK) was transiently transfected into CAOV3 and OV90 cells stably transfected with shTTK2. **E** Western blot assay was used to assess the expression of the TTK protein 48 h after the transfection of the PCMV-TTK plasmid. **F** The effect of TTK overexpression on the proliferation of TTK-silenced CAOV3 and OV90 cells was detected using the MTT assay. **G** Colony formation assays were performed to assess the colony formation capacity when TTK was overexpressed in TTK knockdown ovarian cancer cells (data are mean ± SEM, ^#^*P* > 0.05, *P < 0.05, **P < 0.01, *n* = 3).

We depleted the expression of TTK in CAOV3 and OV90 cells to investigate the potential function of TTK in ovarian cancer (Fig. 2A and Supplementary Fig. S2A, B). The proliferation of ovarian cancer cells with TTK knockdown was significantly decreased (Fig. 2B). Colony formation assays revealed that TTK silencing inhibited the long-term multiplication capacity of CAOV3 and OV90 cells (Fig. 2C and Supplementary Fig. S2C). TTK is a serine/threonine kinase that modulates accurate cell cycle progression. We analyzed the cell cycle to explore whether the inhibitory effect on proliferation was caused by a perturbed cell cycle. As shown in Fig. 2D and Supplementary Fig. S2D, downregulation of TTK obviously decreased the number of cells in G1 phase and increased the number of cells in G2/M phase. In addition, the number of cells in post G2 phase, which indicated multinucleation, was increased in the TTK knockdown group. Numbers of apoptotic cells were increased following TTK depletion (Supplementary Fig. S2E). All the results described above indicated that genetic depletion of TTK significantly suppressed the proliferation of ovarian cancer cells by disturbing cell cycle progression.

We overexpressed TTK in TTK knockdown (shTTK2) cells to further determine the effect of TTK on cell proliferation. The knockdown and overexpression efficiency were verified by RT–qPCR and western blotting. The results revealed that TTK was successfully overexpressed in TTK knockdown cells (Fig. 2E and Supplementary Fig. S2F, G). MTT and colony formation assays were performed to examine the effect of TTK on the proliferation of these cells, and the results indicated that the restoration of TTK in TTK knockdown cells partially rescued the inhibitory effect on cell growth (Fig. 2F, G). According to the aforementioned results, TTK is important for the proliferation of ovarian cancer cells.

### TTK inhibitors impede ovarian cancer proliferation in vitro

**Fig. 3:**
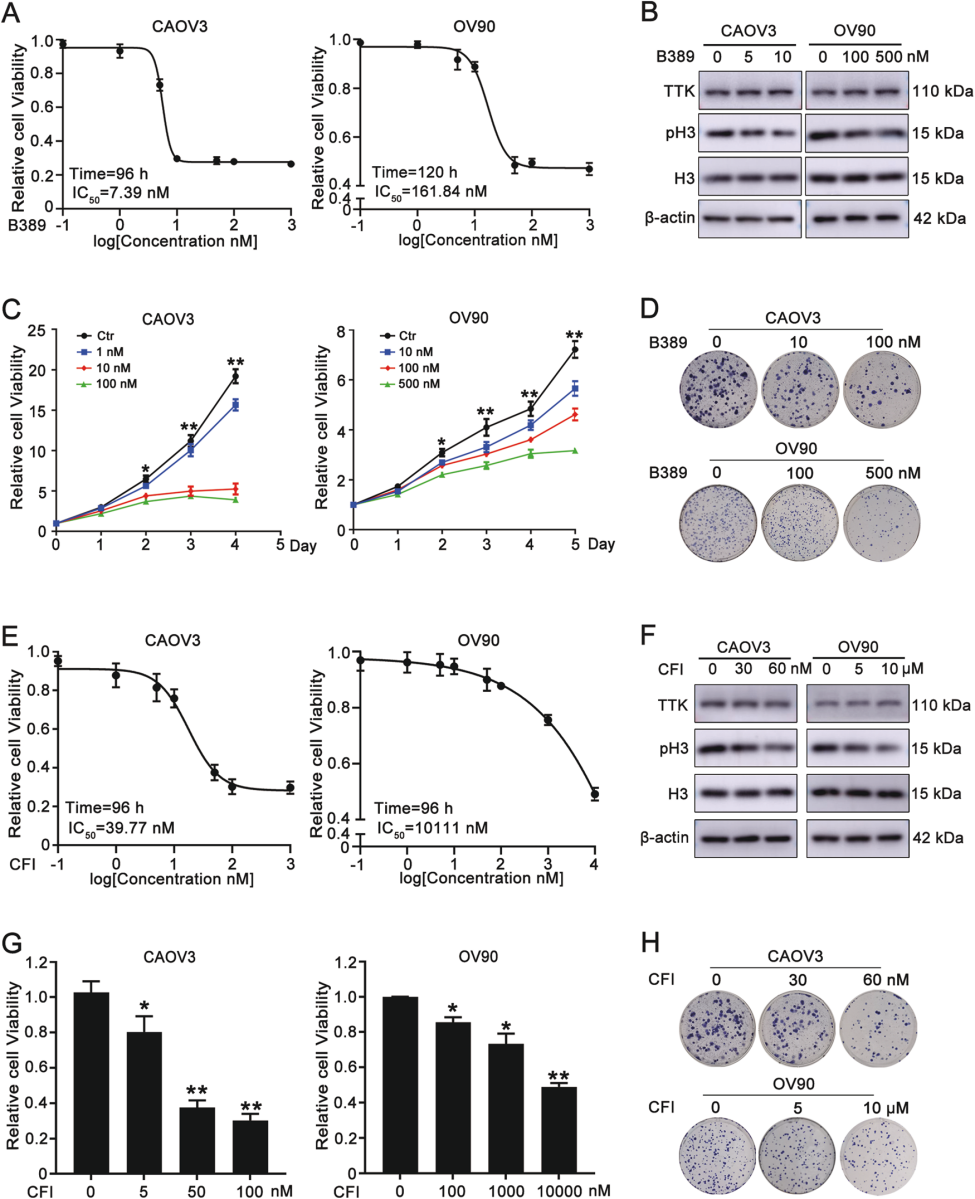
TTK inhibitors impede ovarian cancer proliferation in vitro. **A** CAOV3 and OV90 cells (3 × 10^3^ cells per well of a 96-well plate) were incubated with a series of B389 concentrations (0, 0.1, 1, 5, 10, 50, 100, or 1000 nM) for 96 and 120 h, respectively. MTT assays were performed to determine the IC_50_ of B389 in CAOV3 (IC_50_ = 7.39 nM, 96 h) and OV90 (IC_50_ = 161.84 nM, 120 h) cells. **B** CAOV3 and OV90 cells were treated with different concentrations of B389 (0, 5, or 10 nM for CAOV3 cells and 0, 100, or 500 nM for OV90 cells) for 96 h. The dose-dependent effect of B389 on protein levels of TTK, H3, pH3 (ser10) and β-actin was detected using western blotting. **C** CAOV3 and OV90 cells (1.5 × 10^3^ cells per well of a 96-well plate) were cultured with different concentrations of B389 (0, 1, 10, or 100 nM for CAOV3 cells and 0, 10, 100, or 500 nM for OV90 cells). Dose- and time-dependent effects of B389 on the proliferation of CAOV3 and OV90 cells were detected using MTT assays. **D** First, 1.5 × 10^3^ cells were seeded in each well of a six-well plate, different concentrations of B389 (0, 10, or 100 nM for CAOV3 cells and 0, 100, or 500 nM for OV90 cells) were added after 10 days culture, and cells were treated for 4 days. The effect of B389 on the colony formation ability of ovarian cancer cells was detected. **E**. CAOV3 and OV90 cells were incubated with series concentrations of CFI (0, 0.1, 1, 5, 10, 50, 100, 1000, or 10,000 nM) for 96 h. MTT assays were performed to determine the IC_50_ of CFI in CAOV3 (IC_50_ = 39.77 nM) and OV90 (IC_50_ = 10111 nM) cells. **F** CAOV3 and OV90 cells were treated with different concentrations of CFI (0, 30, or 60 nM for CAOV3 cells and 0, 5, or 10 μM for OV90 cells) for 96 h. Levels of the TTK, H3, pH3 (ser10) and β-actin proteins were detected using western blotting after CFI treatment. **G** The antiproliferative effect of CFI (0, 5, 50, or 100 nM for CAOV3 cells and 0, 100, 1000, or 10,000 nM for OV90 cells) on CAOV3 and OV90 cells was detected using MTT assays. **H** CFI treatment (0, 30, or 60 nM for CAOV3 cells and 0, 5, or 10 μM for OV90 cells) inhibited the colony formation ability of ovarian cancer cells (data are mean ± SEM, *P < 0.05, **P < 0.01, *n* = 3).

BAY1217389 (hereafter named B389), the only TTK inhibitor that had completed a phase I clinical trial, was used to inhibit the function of TTK and further confirm the effect of TTK on cell proliferation. First, a series of concentrations of B389 was added, and an MTT assay was performed to determine the IC_50_ in CAOV3 and OV90 cells (Fig. 3A). The IC_50_ value of B389 was 7.39 nM (96 h) for CAOV3 cells and 161.84 nM (120 h) for OV90 cells (Fig. 3A). Western blot assays were performed to determine the effect of B389 on inhibiting TTK. As shown in Fig. 3B, the protein level of phospho-histone 3 (Ser10), a marker of functional TTK [15], was significantly decreased while the level of TTK did not change, indicating an effect of B389 on interfering with the SAC due to the inhibition of TTK. A dose- and time-dependent inhibitory effect of B389 on the growth of CAOV3 and OV90 cells was observed (Fig. 3C). The colony formation ability of ovarian cancer cells was distinctly suppressed by treatment with B389 (Fig. 3D). Similar to genetic inhibition of TTK, B389 treatment caused a decrease in the number of cells in G1 phase and an increase in the number of cells in G2/M phase of the cell cycle (Supplementary Fig. S3A). In addition, B389 significantly increased the percentage of apoptotic ovarian cancer cells (Supplementary Fig. S3B).

An additional TTK inhibitor, CFI-402257 (hereafter called CFI), was used to verify the antiproliferative effect of TTK inhibitors. As shown in Fig. 3E–H, TTK inhibition by CFI also resulted in slower growth of ovarian cancer cells.

These results implied that TTK inhibitors effectively inhibited ovarian cancer cell proliferation in vitro.

### TTK silencing enhances cisplatin sensitivity in ovarian cancer cells

**Fig. 4:**
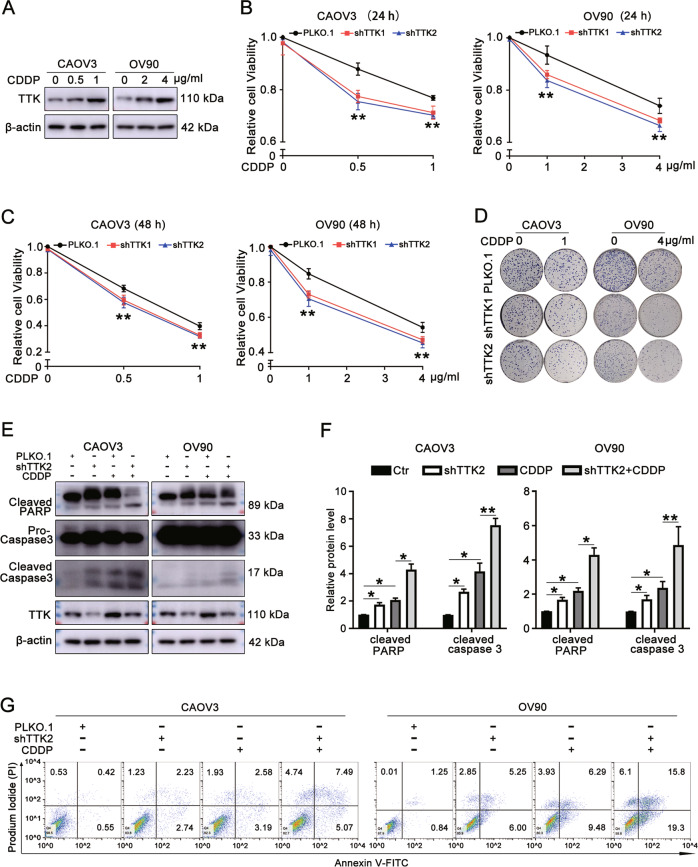
TTK silencing enhances cisplatin sensitivity in ovarian cancer cells. **A** Western blotting was performed to detect the TTK protein level in CAOV3 and OV90 cells treated with different concentrations of cisplatin (0, 0.5, or 1 μg/ml for CAOV3 and 0, 2, or 4 μg/ml for OV90) for 48 h. **B**−**D** CAOV3 and OV90 cells stably transfected with PLKO.1 or the TTK shRNA (shTTK1 and shTTK2) were treated with different concentrations of CDDP (0, 0.5, or 1 μg/ml for CAOV3 and 0, 1, or 4 μg/ml for OV90). The MTT assay was performed at 24 h (**B**) and 48 h (**C**) and the colony formation assay (**D**) was performed to evaluate the effect of TTK on CDDP sensitivity. **E**−**G** CAOV3 and OV90 cells stably transfected with PLKO.1 or shTTK2 were treated with 2 or 4 μg/ml CDDP for 48 h. **E**, **F** Western blot assays were conducted to assess the relative protein levels of cleaved PARP, cleaved caspase-3, and TTK normalized to β-actin. **G** Flow cytometry was used to detect apoptotic cells (data are mean ± SEM, *P < 0.05, **P < 0.01, *n* = 3).

CAOV3 and OV90 cells were treated with cisplatin (hereafter referred to as CDDP) for 48 h, and western blot assays showed an increase level of the TTK protein following CDDP treatment (Fig. 4A). CAOV3 and OV90 cells stably transfected with shTTK or the control vector PLKO.1 were incubated with a series of concentrations of CDDP. MTT assays revealed that TTK silencing significantly enhanced the sensitivity of ovarian cancer cells to CDDP at 24 and 48 h (Fig. 4B, C). The colony formation assay produced similar results (Fig. 4D). In addition, the protein levels of cleaved PARP and cleaved caspase-3 were increased following TTK knockdown, and the increases were more significant after combination treatment with CDDP (Fig. 4E, F). The flow cytometry assay also indicated that TTK silencing enhanced CDDP-induced apoptosis (Fig. 4G). Taken together, TTK silencing enhances CDDP-induced apoptosis and increases the sensitivity of ovarian cancer cells to CDDP.

### TTK inhibitors render CAOV3 and OV90 cells more sensitive to cisplatin

**Fig. 5:**
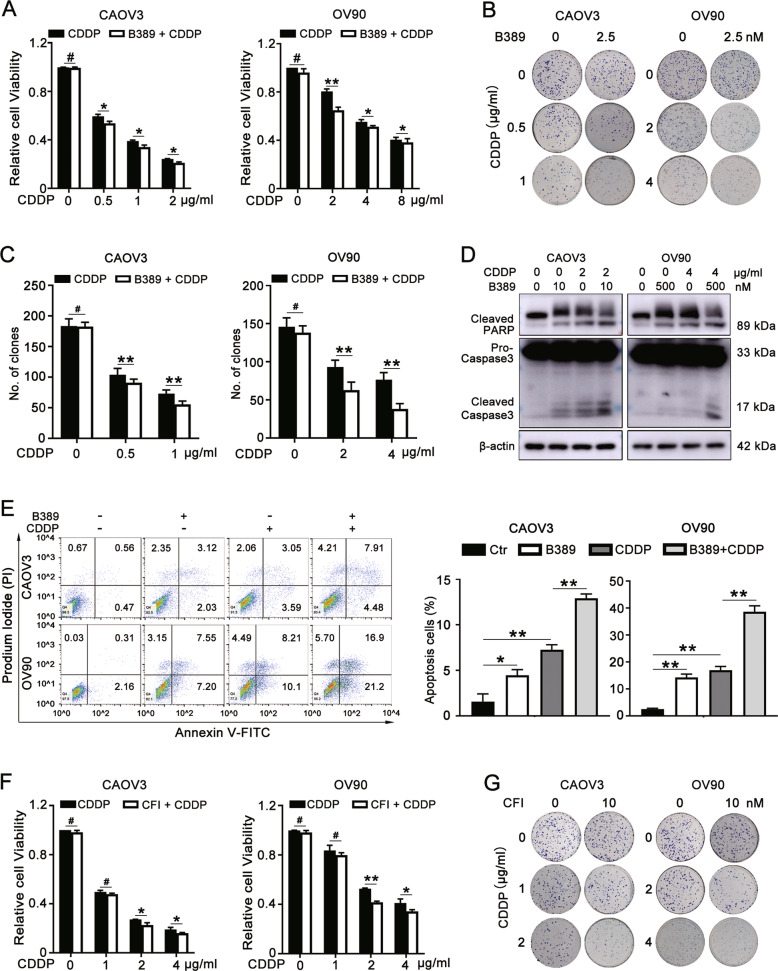
TTK inhibitors render CAOV3 and OV90 cells more sensitive to cisplatin. **A**−**C** CAOV3 and OV90 cells were treated with the indicated concentrations of CDDP (0, 0.5, 1, or 2 μg/ml for CAOV3 cells and 0, 2, 4, or 8 μg/ml for OV90 cells) in combination with or without B389 (2.5 nM) for 48 h. **A** The MTT assay was conducted to determine cell viability. **B**, **C** The cotreatment efficiency of B389 and CDDP was assessed using the colony formation assay. **D**, **E** CAOV3 and OV90 cells were treated with DMSO, B389 (10 nM for CAOV3 and 500 nM for OV90), or CDDP (2 μg/ml for CAOV3 and 4 μg/ml for OV90) alone or in combination with B389 and CDDP. **D** The protein levels of cleaved PARP, cleaved caspase-3, and TTK were evaluated using western blotting. β-actin served as an endogenous control. **E** Apoptotic cells in different groups were detected using flow cytometry. **F**, **G** The MTT assay (**F**) and colony formation assay (**G**) were evaluated after treatment with 0, 1, 2, 4 μg/ml cisplatin and/or 10 nM CFI (data are mean ± SEM, ^#^*P* > 0.05, *P < 0.05, **P < 0.01, *n* = 3).

We further investigated whether a TTK inhibitor also increased the sensitivity of ovarian cancer to cisplatin. CAOV3 and OV90 cells were treated with the indicated concentrations of CDDP alone or in combination with B389 for 48 h. The drug doses applied for chemosensitization studies were approximately half of the IC_50_ value to exclude the antiproliferative effect of B389. The MTT assay (Fig. 5A) and colony formation assay (Fig. 5B, C) revealed that the lethal effect of the combination treatment on ovarian cancer cells was more distinct than that of the single treatment alone. Western blot assays showed that B389 or CDDP treatment alone increased the levels of apoptosis-related proteins, and this change was more remarkable when the combination treatment was applied (Fig. 5D). The flow cytometry assay also showed that the combination treatment increased the number of apoptotic cells (Fig. 5E). TTK inhibition with CFI also increased the sensitivity to cisplatin (Fig. 5F, G). In conclusion, TTK inhibitors render CAOV3 and OV90 cells more sensitive to cisplatin.

### NGS analysis of the signaling pathways affected by TTK knockdown

Next-generation sequencing (NGS) was carried out in A2780 cells transfected with siTTK2 or NC (*n* = 3) to clarify the potential mechanism by which TTK depletion suppressed the proliferation and increased the sensitivity of ovarian cancer cells to cisplatin. As shown in Supplementary Figs. S4A, 554 upregulated and 579 downregulated DEGs were identified. The biological process (BP) and KEGG analyses of the downregulated genes showed that TTK knockdown affected DNA replication and the cell cycle pathways (Supplementary Fig. S4B, C), consistent with previous studies, indicating that the sequencing results were reliable. The autophagy pathway, one of the top five pathways affected by TTK depletion, has been shown to be involved in cisplatin resistance in ovarian cancer. Few studies have focused on the direct relationship between TTK and autophagy, although TTK has been reported to be involved in the PI3K/AKT or Akt/mTOR pathways, which regulate autophagy. Consequently, we speculated that TTK inhibition suppresses ovarian cancer cell proliferation and increases the sensitivity of ovarian cancer cells to cisplatin by inhibiting the autophagy pathway.

### TTK depletion inhibits autophagy by activating the mTOR signaling pathway in ovarian cancer cells

**Fig. 6:**
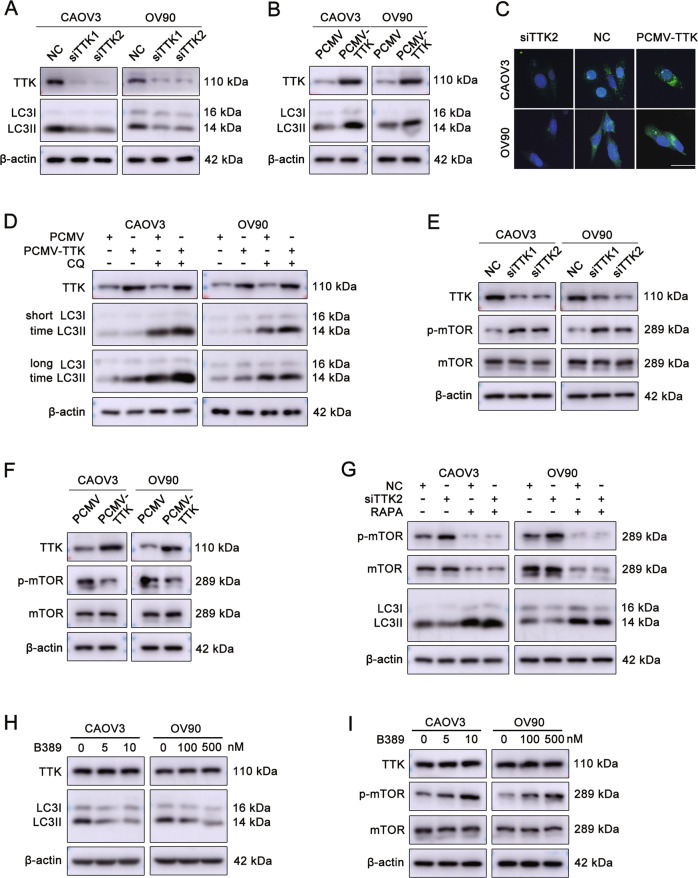
TTK depletion inhibits autophagy by activating the mTOR signaling pathway in ovarian cancer cells. **A** CAOV3 and OV90 cells were transiently transfected with NC or siTTK for 48 h. Protein levels of LC3-I, LC3-II, TTK, and β-actin were assessed using western blotting. **B** Western blot assays were conducted to detect the LC3-I, LC3-II, TTK and β-actin protein levels in CAOV3 and OV90 cells after transfection with PCMV or PCMV-TTK. **C** Representative images of immunofluorescence staining for LC3B in ovarian cancer cells transfected with siTTK, NC, and PCMV-TTK for 48 h (×400). Scale bar: 15 µm. **D** CAOV3 and OV90 cells transfected with PCMV or PCMV-TTK were treated with or without CQ (50 μM) for 24 h. Levels of the LC3-I, LC3-II, TTK and β-actin proteins were analyzed using western blotting. **E** CAOV3 and OV90 cells were transfected with NC or siTTK for 48 h. Levels of the p-mTOR, mTOR, TTK, and β-actin proteins were evaluated using western blotting. **F** Western blot assays were performed to show the levels of the p-mTOR, mTOR, TTK and β-actin proteins in CAOV3 and OV90 cells transfected with PCMV or PCMV-TTK for 48 h. **G** CAOV3 and OV90 cells transfected with NC or siTTK were treated with or without 100 nM rapamycin for 24 h. The levels of p-mTOR, mTOR, LC3-I/II, TTK, and β-actin were measured using western blotting. **H** Levels of the LC3-I, LC3-II, TTK, and β-actin proteins in CAOV3 and OV90 cells treated with B389 for 48 h. **I** Western blot analysis was performed to detect the levels of the p-mTOR, mTOR, TTK and β-actin proteins in CAOV3 and OV90 cells treated with B389 for 48 h (data are mean ± SEM, *n* = 3).

Additional experiments were performed to further confirm that TTK is involved in the autophagy pathway. TTK silencing decreased the level of the LC3-II protein (Fig. 6A and Supplementary Fig. S5A), while TTK overexpression increased LC3-II levels in CAOV3 and OV90 cells (Fig. 6B and Supplementary Fig. S5B). In addition, immunofluorescence staining showed that TTK knockdown reduced the number of LC3B puncta and TTK overexpression increased LC3B accumulation (Fig. 6C and Supplementary Fig. S5C). The level of LC3-II was increased to a greater extent following chloroquine (CQ) treatment, suggesting that TTK induced autophagy in ovarian cancer cells (Fig. 6D and Supplementary Fig. S5D). Meanwhile, TTK knockdown increased the level of phosphorylated mTOR (p-mTOR), whereas TTK overexpression exerted the opposite effect (Fig. 6E, F and Supplementary Fig. S5E, F). Furthermore, rapamycin treatment of cells transfected with siTTK partly rescued the effect of TTK knockdown on LC3-II and p-mTOR levels (Fig. 6G and Supplementary Fig. S5G). B389 treatment also decreased the level of LC3-II and increased p-mTOR levels (Fig. 6H, I and Supplementary Fig. S5H, I). Thus, TTK depletion suppresses autophagy in ovarian cancer cells by activating the mTOR signaling pathway.

### TTK silencing increases the sensitivity of ovarian cancer to cisplatin through the mTOR/autophagy pathway

**Fig. 7:**
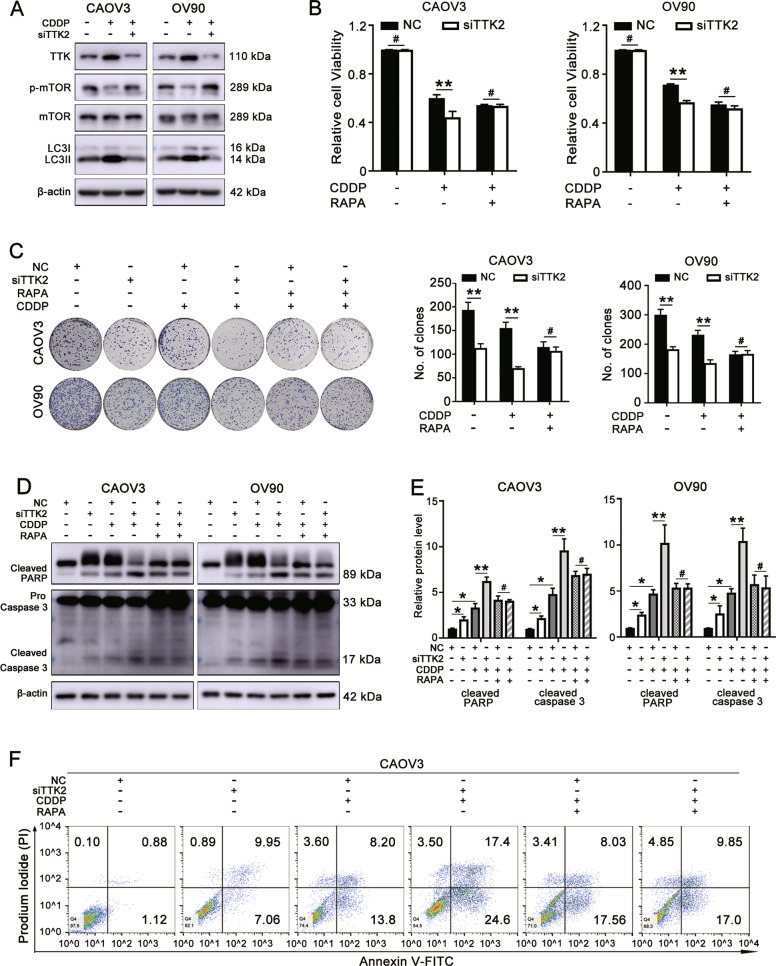
TTK silencing increases the sensitivity of ovarian cancer to cisplatin through the mTOR/autophagy pathway. **A** CAOV3 and OV90 cells transfected with NC or siTTK2 were treated with 1 or 2 μg/ml CDDP for 48 h. The relative protein levels of p-mTOR, mTOR, LC3-I/II and TTK were detected using western blotting. **B**, **C** CAOV3 and OV90 cells with or without TTK knockdown were treated with 1 or 2 μg/ml CDDP alone or in combination with 100 nM rapamycin. Cell viability (**B**) and the colony formation ability (**C**) were detected. **D**−**F** CAOV3 and OV90 cells transfected with NC or siTTK2 were treated with 2 or 4 μg/ml CDDP for 24 h and then treated in combination with or without 100 nM rapamycin for 24 h. **D**, **E** The protein levels of cleaved PARP and cleaved caspase-3 were evaluated using western blotting. **F** Flow cytometry was performed to detect apoptotic cells (data are mean ± SEM, ^#^*P* > 0.05, *P < 0.05, **P < 0.01, *n* = 3).

As shown above, TTK knockdown inhibited autophagy and increased sensitivity to cisplatin; therefore, we further explored whether TTK silencing enhanced the cytotoxicity of cisplatin by suppressing autophagy. TTK inhibition decreased the increase in the LC3-II level induced by cisplatin (Fig. 7A). Meanwhile, CDDP treatment decreased the p-mTOR level, and TTK depletion partially reversed the inhibition of p-mTOR induced by CDDP (Fig. 7A). In addition, CAOV3 and OV90 cells transfected with siTTK2 or NC were incubated with CDDP alone or in combination with the mTOR pathway inhibitor rapamycin. The downregulation of TTK increased the sensitivity of ovarian cancer cells to CDDP, and this effect was partially reversed by rapamycin (Fig. 7B, C). As shown in Fig. 7D−F, rapamycin treatment decreased apoptosis in the group treated with the combination of cisplatin and TTK knockdown. These results implied that TTK silencing increases the cisplatin sensitivity of ovarian cancer by activating mTOR signaling to inhibit autophagy.

### Knockdown of TTK increases cisplatin sensitivity by inhibiting autophagy in vivo

**Fig. 8:**
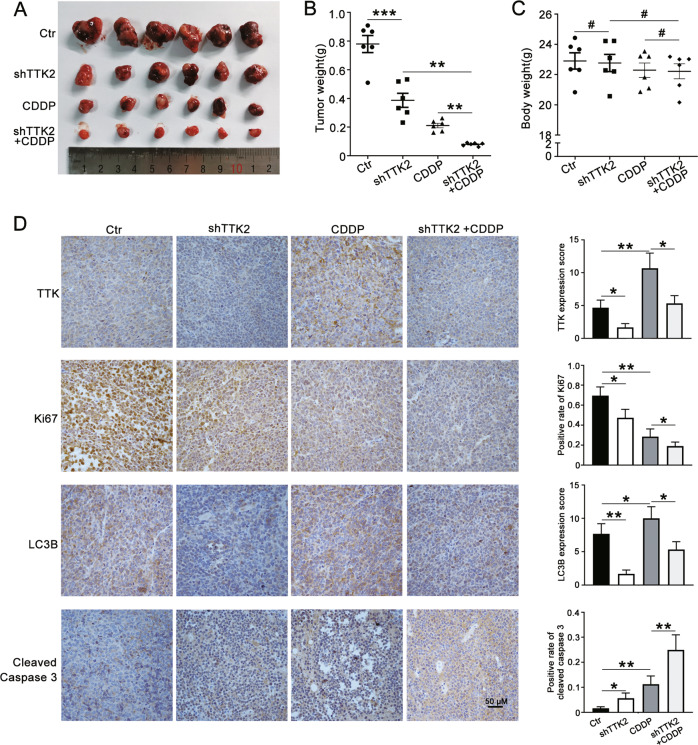
Knockdown of TTK increases cisplatin sensitivity by inhibiting autophagy in vivo. CAOV3 cells transfected with shTTK2 or PLKO.1 were subcutaneously injected into the left armpit of each 5-week-old female mouse. When the tumor volumes were approximately 100 mm^3^, each group of mice was randomly divided into two subgroups and treated with or without CDDP (2 mg/kg) for 14 days. **A** Photographs of tumors from each group. **B** The tumor weights in each group. **C** The bodyweight of each group in (**A**). **D** Representative images of IHC staining of TTK, Ki67, LC3B, and cleaved caspase-3 in tumor tissues (×400). Scale bar: 50 µm (data are mean ± SEM, ^#^*P* > 0.05, *P < 0.05, **P < 0.01, ***P < 0.001, *n* = 6).

The role of TTK knockdown in vivo was also investigated. As shown in Fig. 8A, B, TTK knockdown or cisplatin treatment alone reduced the tumor volume, and the combination treatment led to a more obvious decrease in the tumor volumes and tumor weight. Meanwhile, the bodyweight had did not display evident changes during therapy (Fig. 8C). IHC staining of the tumor showed that TTK knockdown reduced the levels of Ki67 and LC3B and increased the level of cleaved caspase-3, especially when administered in combination with cisplatin treatment (Fig. 8D). Based on these data, inhibition of TTK suppresses the growth of tumors and increases the cytotoxicity of cisplatin in vivo by inhibiting autophagy.

## Discussion

In this study, we investigated the roles of TTK in the progression and cisplatin resistance of ovarian cancer. TTK expression was obviously upregulated in HGSOC and cisplatin-resistant ovarian cancer cells (Fig. 1). Genetic (shRNA and siRNA) and pharmacological (BAY1217389 and CFI-402257) inhibition of TTK suppressed proliferation and enhanced chemosensitization in ovarian cancer cells (Figs. 2–5). We discovered that TTK knockdown or inhibition blocked autophagy (Fig. 6). TTK silencing enhanced the cytotoxicity of cisplatin partially through the mTOR/autophagy pathway (Fig. 7). In vivo, TTK knockdown inhibited tumor growth and increased cisplatin sensitivity by inhibiting the autophagy pathway (Fig. 8). In conclusion, inhibition of TTK combined with cisplatin is a potentially effective therapy to overcome the insensitivity or resistance to cisplatin in patients with ovarian cancer.

In two independent GEO datasets, we identified DEGs correlated with ovarian cancer progression, and a KEGG analysis of the DEGs revealed the specific enrichment of the cell cycle pathway. TTK was the only gene in the cell cycle pathway with clinical-grade inhibitors that has not been investigated in ovarian cancer. Many other genes in the cell cycle pathway, such as CDC7, CDK1 and CHEK1, have previously been reported to be correlated with the proliferation and chemoresistance of ovarian cancer [41–43]. Thus, our unbiased process to identify novel genes involved in ovarian cancer formation was rational and effective. Then, we searched two independent cisplatin resistance datasets and found that TTK expression was increased in cisplatin-resistant ovarian cancer cells. These results implied that TTK might be involved in the tumorigenesis and chemoresistance of ovarian cancer.

TTK, a regulator of SAC, plays an important role in cell cycle progression [44–48]. TTK inhibition has been reported to result in irregular mitosis, increased aneuploidy, and mitotic catastrophe [49, 50]. In our study, genetic and pharmacological inhibition of TTK caused a disordered cell cycle and increased the number of cells in the post G2 phase. In addition, TTK has been discovered to function as an oncogene in various cancers [12–14, 18–21], and TTK inhibition has already been studied as a targeted therapy for breast cancer, glioblastoma, liver cancer and other tumors [15, 16, 22, 33]. Similar to previous studies, our results identified TTK as an oncogene in ovarian cancer, and inhibition of TTK suppressed the proliferation of ovarian cancer in vitro and in vivo. Furthermore, we indicated that TTK inhibition is an effective strategy to increase cisplatin sensitivity in ovarian cancer.

We performed an NGS analysis to determine the underlying mechanism by which TTK inhibition suppressed the growth of ovarian cancer and found that the expression of genes involved in the autophagy pathway was decreased after TTK knockdown. Autophagy plays a crucial role in the oncogenesis, progression, and treatment of various cancers [51, 52]. However, few studies have focused on the direct relationship between TTK and autophagy. TTK inhibits the PI3K/AKT pathway, and TTK knockdown inhibits the PKCα/ERK signaling pathway in colon cancer [20]. Huang et al. [19] and Liu et al. [14] found that TTK activates the Akt/mTOR pathway in gastric and liver cancer. All the aforementioned signaling pathways are involved in regulating autophagy; thus, we focused on the regulatory relationship between TTK and autophagy in ovarian cancer. Our results revealed that TTK inhibition decreased autophagy in ovarian cancer cells.

The mTOR protein, a component of the classical pathway regulating autophagy, is a serine/threonine kinase [53]. We speculated that TTK inhibition decreases autophagy through the mTOR pathway in ovarian cancer. TTK knockdown activated the mTOR pathway and the administration of rapamycin to inhibit mTOR partially rescued the elevated p-mTOR level in our study. Thus, TTK inhibition suppressed the progression of ovarian cancer by activating mTOR and further decreasing autophagy.

According to previous studies, autophagy contributes to cisplatin resistance in ovarian cancer [54–56]. In the present study, cisplatin treatment induced TTK expression and activated autophagy. Genetic and pharmacological inhibition of TTK decreased autophagy and increased sensitivity to cisplatin. In addition, inhibiting mTOR by rapamycin in TTK knockdown cells partially reversed the increased cisplatin sensitivity induced by TTK downregulation. Therefore, the cisplatin-triggered upregulation of TTK decreased the cisplatin sensitivity of ovarian cancer through the mTOR/autophagy pathway.

The insensitivity or resistance to cisplatin is still a troublesome issue in HGSOC treatment. As shown in the present study, TTK functions as an oncogene in HGSOC progression. TTK inhibitors, which have been investigated and tested in clinical trials, resulted in increased apoptosis induced by cisplatin and increased sensitivity to cisplatin in vitro and in vivo. Based on these findings, the use of TTK inhibitors in combination with cisplatin may lead to improved cisplatin response rates in patients with HGSOC presenting high TTK expression. In summary, our study may provide a theoretical foundation for the use of combination therapy composed of cisplatin and TTK inhibitors as an HGSOC treatment in the future.

## Materials and methods

### Bioinformatics analysis

Two independent datasets (GSE14407 and GSE10971) in the GEO database, including the gene expression profiles of primary serous ovarian cancer and normal control tissues, were downloaded. The DEGs with log2 (fold change) > 1 and *P* value < 0.001 were analyzed with the DESeq R package. Then, the DEGs in each separate dataset were compared, and the overlapping genes were investigated further. The KEGG pathways of the overlapping DEGs were analyzed using the DAVID website (https://david.ncifcrf.gov/tools.jsp). GSE15709 and GSE98559 were used to assess the difference in TTK expression between cisplatin-resistant and cisplatin-sensitive ovarian cancer cells. The expression of the TTK mRNA in ovarian cancer and corresponding normal tissues in the GEPIA (http://gepia.cancer-pku.cn/) and Oncomine (www.oncomine.org) databases was retrieved. Kaplan–Meier plotter (http://kmplot.com/analysis/) was used to analyze survival based on TTK expression.

### Patients and tissue samples

The formalin-fixed and paraffin-embedded tissues and fresh-frozen tissues included in this study were collected from Qilu Hospital, Shandong University. Patients or their guardians provided written informed consent before surgery, and ethical approval was obtained from the Ethics Committee at Qilu Hospital of Shandong University (KYLL-2020(KS)-131). Malignant tissue samples were obtained from patients diagnosed with primary HGSOC who underwent surgery. Normal fallopian tube (FT) tissues were regarded as normal controls and were obtained from women with benign uterus or adnexal diseases who underwent hysterectomy and bilateral salpingo-oophorectomy.

### Cell lines and cell culture

CAOV3 and OV90 cell lines were purchased from American Type Culture Collection (ATCC, Manassas, VA, USA). The HEK293T cell line was obtained from the Chinese Academy of Sciences (Shanghai, China). CAOV3 and OV90 cells were cultured in Dulbecco’s modified Eagle’s medium (DMEM) supplemented with 15% fetal bovine serum (FBS), while HEK293T cells were maintained in DMEM supplemented with 10% FBS (all from Gibco, Grand Island, NY, USA). A humidified 37 °C incubator with 5% CO_2_ was used to culture all cells. Short tandem repeat (STR) profiling was performed to confirm the cells and to exclude mycoplasma contamination.

### RNA isolation and RT–qPCR

TRIzol reagent (15596018, Invitrogen) was used to extract the total RNA from cultured cells and tissues. Total RNA was reverse transcribed to cDNA templates using the protocol of the PrimeScript RT Reagent Kit (RR037A, TaKaRa, Kyoto, Japan). Real-time quantitative PCR (RT–qPCR) was performed according to the instructions of SYBR Premix Ex Taq (RR420A, TaKaRa) using the 7900HT Fast Real-Time PCR System (Applied Biosystems, Waltham, MA, USA). β-actin served as an endogenous control. The comparative Ct (2-ΔΔCt) method was used to analyze the raw data. The sequences for the primer pairs were as follows: TTK forward primer: CCGAGATTTGGTTGTGCCTGGA; reverse primer: CATCTGACACCAGAGGTTCCTTG; and β-actin forward primer: CACCATTGGCAATGAGCGGTTC; reverse primer: AGGTCTTTGCGGATGTCCACGT.

### Protein extraction and western blotting

Tissues and cells were lysed in RIPA buffer (Beyotime Institute of Biotechnology, China) supplemented with PMSF (1%) and NaF (1%). The lysate was harvested and centrifuged at 4 °C before the supernatants were collected. The BCA Protein Assay kit (Merck Millipore, USA) was employed to quantify the protein concentration. Equivalent proteins were loaded on gels and separated by sodium dodecyl sulfate polyacrylamide gel electrophoresis (SDS–PAGE) followed by transfer to PVDF membranes (Merck Millipore, Burlington, MA, USA). PVDF membranes were incubated with specific primary antibodies at 4 °C for at least 14 h before the indicated secondary antibodies were added. After a 1 h incubation with the secondary antibodies, an enhanced chemiluminescence detection kit (ECL ORT2655, PerkinElmer, Waltham, MA, USA) was used to detect the signal with a GE Amersham Imager 600 (GE). β-actin was used as an endogenous control. The relative protein level was quantitatively compared to β-actin using ImageJ 1.52a software (US National Institutes of Health).

### IHC

Four-micrometer sections of formalin-fixed and paraffin-embedded tissues were used to perform immunohistochemical (IHC) staining. Tissue sections were deparaffinized and rehydrated in xylene and graded concentrations of ethanol, respectively. EDTA buffer and 3% hydrogen peroxide were used for antigen retrieval and quenching endogenous peroxidase activity, respectively. Then, the indicated primary antibody was incubated with sections in a humid chamber overnight at 4 °C. The DAB (ZSGB-BIO, Beijing, China) detection system was used to visualize the staining after a 1 h incubation with secondary antibody.

### Antibodies and reagents

Antibodies against TTK (ab11108) and LC3B (ab192890) were purchased from Abcam (Cambridge, UK), the antibody against p-mTOR (5536T) was obtained from Cell Signaling Technology (Danvers, MA, USA); the antibody against mTOR (GB11405) was purchased from Servicebio (Wuhan, China), and antibodies against β-actin (A5441), CQ (C6628) and CDDP (PHR1624) were purchased from Sigma-Aldrich (St. Louis, MO, USA). Rapamycin (S1039), BAY1217389 (S8215) and paclitaxel (S1150) were purchased from Selleck Chemicals (Houston, TX, USA). CFI-402257 was purchased from GlpBio (Shanghai, China).

### Plasmid construction, lentivirus production and siRNA transfection

The shRNA primer pairs for TTK were obtained from GenePharma (Shanghai, China) and were inserted into a PLKO.1 vector to generate an shTTK plasmid. The TTK overexpression plasmid was obtained from GeneChem (Shanghai, China), and the open reading frame of TTK was copied and cloned into the PCMV vector to obtain the PCMV-TTK plasmid. PLKO.1 and PCMV vector served as mock controls. HEK293T cells at an appropriate confluence were cotransfected with psPAX2, pMD2.G, and PLKO.1/shTTK or PCMV/PCMV-TTK plasmids to produce the corresponding lentivirus. CAOV3 and OV90 cells were transfected with the indicated lentivirus and selected with 2 μg/ml puromycin (Merck Millipore, USA) for 5–7 days to obtain stably transfected cells. The sequences of short hairpin RNA (shRNA) were as follows: shTTK1: 5′-AATGAACAAAGTGAGAGACAT-3′ and shTTK2: 5′-GCACAATTTGAACTGTCACAA-3′.

Specific TTK siRNAs (siTTK1: 5′-UGAACAAAGUGAGAGACAUTT-3′; siTTK2: 5′-GGAUUUAAGUGGCAGAGAATT-3′) and a negative control siRNA (NC: 5′-UUCUCCGAACGUGUCACGUTT-3′) were purchased from GenePharma (Shanghai, China). Cancer cells at an appropriate confluence were transfected with siTTK or NC using Lipofectamine 2000 according to the manufacturer’s instructions (11668-019, Invitrogen).

### Cell proliferation and colony formation assays

For the cell proliferation assay, 1500 cells were seeded in each well of a 96-well plate and incubated with the corresponding culture medium for the indicated period. Cell proliferation was detected using the MTT assay. Twenty microlitres of MTT (Sigma-Aldrich, USA, 0.5 mg/ml) were added to each well. After 4 h of continuous incubation, the supernatant was discarded, and 100 μl of DMSO (Sigma-Aldrich, USA) was added. Ten minutes later, the absorbance value at 490 nm was detected using a Varioskan Flash microplate reader (Thermo Scientific).

For the colony formation assay, 1500 cancer cells were cultivated in each well of six-well plates for 1–2 weeks, and the medium was changed every 3 days. When the colonies contained more than 50 cells, the colonies were fixed with methanol and stained with 0.6% crystal violet.

### Drug treatment

Drug resistance was analyzed with the MTT assay and clonogenic assays mentioned above. A total of 3 × 10^3^ and 1.5 × 10^3^ cells were seeded into each well of 96-well plates and 6-well plates, respectively. Then, the cells were treated with specific concentrations of the indicated drugs for the indicated periods.

### Cell cycle and apoptosis assays

For the cell cycle assay, treated cells were collected, washed and stained with propidium iodide (PI) for 30 min according to the manufacturer’s instructions (CCS012, MultiSciences Biotech Co., Ltd.). Then, a flow cytometer (FACSCalibur, BD, USA) was used to analyze the cell cycle phase distribution of different groups, and Modifit LT software was applied to analyze the results.

For the apoptosis assay, differently treated cells were harvested, rinsed, resuspended in 1× binding buffer (556547, BD Bioscience, Franklin Lakes, NJ, USA) and stained with FITC Annexin V and PI according to the manufacturer’s protocol. Stained cells were analyzed using a flow cytometer (FACSCalibur, BD, USA).

### Immunofluorescence staining

Cells pre-seeded on 24 coverslips were fixed with 4% paraformaldehyde, blocked with normal goat serum, and LC3B (ab192890, dilution 1:100) antibody was added and incubated for 16 h at 4 °C. Then, the appropriate secondary antibody (donkey anti-rabbit IgG Alexa Fluor-488 1:150; Invitrogen, Waltham, MA, USA) was added and incubated for another 1 h. After counterstaining the nuclei with DAPI, cells were photographed with a Zeiss LSM 780 (Carl Zeiss, Jena, Germany).

### Tumor formation assay in nude mice

The tumor formation assay was performed in a pathogen-free facility with the approval of the Shandong University Animal Care and Use Committee. Approximately 1 × 10^7^ CAOV3 cells transfected with shTTK2 or PLKO.1 were suspended in 150 μl of PBS and subcutaneously injected into the left armpit of each female athymic BALB/c nude mouse (4–5 weeks old; NBRI of Nanjing University, Nanjing, China). The tumor volumes were observed, measured and calculated once every other day. When the tumor volumes were approximately 100 mm^3^, each group of mice was randomly divided into two subgroups and treated with or without CDDP (2 mg/kg) for 14 days. Thus, four groups (6 mice in each group) were analyzed: control, shTTK2, CDDP, and shTTK2+CDDP. At 14 days posttreatment, the mice were sacrificed, and the tumors were detached, photographed and weighed.

### High-throughput differential gene expression analysis

This analysis was conducted to compare A2780 cells transfected with siTTK2 and NC (*n* = 3). Total RNA was extracted 48 h after transfection, and next-generation sequencing was performed by Origingene (Shanghai, China). DEGs refer to genes with a |fold change | (FC) > 1.5 and an adjusted *P* value (FDR) < 0.05. GO enrichment and KEGG pathway analyses of the DEGs were conducted using the DAVID website (https://david.ncifcrf.gov/tools.jsp). The RNA-seq data generated in this study have been deposited in the NCBI GEO database under the accession number GSE176220.

### Statistical analysis

The DEGs in GEO datasets were analyzed with the DESeq R package based on the criteria of a log2 (FC) > 1 and *P* value < 0.001. The KEGG pathway analysis was performed on the DAVID website (https://david.ncifcrf.gov/tools.jsp). Survival curves were estimated using the Kaplan–Meier method on the Kaplan–Meier plotter website (http://kmplot.com/analysis/). For IHC, qPCR, western blot, cellular functions, cell cycle and apoptosis assays, three independent repetitions were conducted, and the data are presented as the means ± SEMs. The data were analyzed using Statistical Product and Service Solutions (SPSS, Inc., Chicago, IL, USA) (22.0) statistical software. Significant differences between groups were analyzed using Student’s *t* test and one-way ANOVA. *P* < 0.05 was considered statistically significant (^#^*P* > 0.05, *P < 0.05, **P < 0.01, and ***P < 0.001). GraphPad Prism 8.00 (GraphPad Software, La Jolla, CA, USA) and Adobe Photoshop CC 2019 (Adobe, San Jose, CA, USA) were the main software programs used to process the images.

## Supplementary information

Supplementary materials

checklist

author contribution

## Data Availability

The datasets used and/or analyzed during the current study are available from the corresponding author on reasonable request.
