# Supplementary material for: TTK inhibition increases cisplatin sensitivity in high-grade serous ovarian carcinoma through the mTOR/autophagy pathway
Source: Cell Death Dis. 2021 Dec 7;12(12):1135. doi: 10.1038/s41419-021-04429-6 (PMC8651821; doi:10.1038/s41419-021-04429-6)
Supplement: Supplementary file 1 — Supplementary materials [file 41419_2021_4429_MOESM1_ESM.docx]

**Supplementary figures and figure legends:**

**Supplementary figure legends：**

Supplementary Figure S1. TTK is higher expressed in cancers than in normal controls.

A-C. The mRNA level of TTK between serous ovarian carcinoma and normal controls from Oncomine and GEPIA databases.

D. The mRNA level of TTK in various cancers.

Supplementary Figure S2. TTK depletion inhibits ovarian cancer cell proliferation by disturbing cell cycle progression.

A. The mRNA level of TTK in CAOV3 and OV90 cells after TTK knockdown.

B. Quantification protein levels in Figure 2A.

C. Quantification of the number of clones in Figure 2C.

D. Quantification of the cell cycle distribution in Figure 2D.

E. Apoptosis assays were performed to assess apoptotic cells in CAOV3 and OV90 cells transfected with PLKO.1 or shTTK.

F. The mRNA level of TTK in CAOV3 and OV90 cells when TTK overexpression plasmid（PCMV-TTK）was transfected to TTK knockdown cells.

G. Quantification protein levels in Figure 2E.

(Data are mean ± SEM, *p < 0.05, **p < 0.01, ****P* < 0.001, n = 3)

Supplementary Figure S3. TTK inhibitor B389 changes the cell cycle distribution and enhances apoptosis of ovarian cancer cells.

A. Flow cytometry was used to detect the cell cycle of CAOV3 and OV90 cells treated with B389.

B. Apoptosis assays exhibited the apoptotic cells following treatment with B389.

(Data are mean ± SEM, *p < 0.05, **p < 0.01, n = 3)

Supplementary Figure S4. NGS analysis of the signaling pathways affected by TTK knockdown.

Next-generation sequencing (NGS) was carried out in A2780 cells transfected with siTTK2 or NC (n=3) to reveal the mRNA expression profile.

A. Volcano plot displayed the DEGs between siTTK and NC groups.

B. Biological Process analysis of downregulated genes in siTTK group compared to NC group based on GO database.

C. The KEGG pathway analysis of the down-regulated DEGs.

Supplementary Figure S5. (C) The proportion of cells with LC3B puncta (> 5) in Figure 6C. Other figures showed the quantification of relative protein levels in Figure 6A-6I.

(Data are mean ± SEM, ^#^P > 0.05, **p* < 0.05, ***p* < 0.01, ****p* < 0.001, n = 3).

**Supplementary figures**

**Supplementary Figure S1**


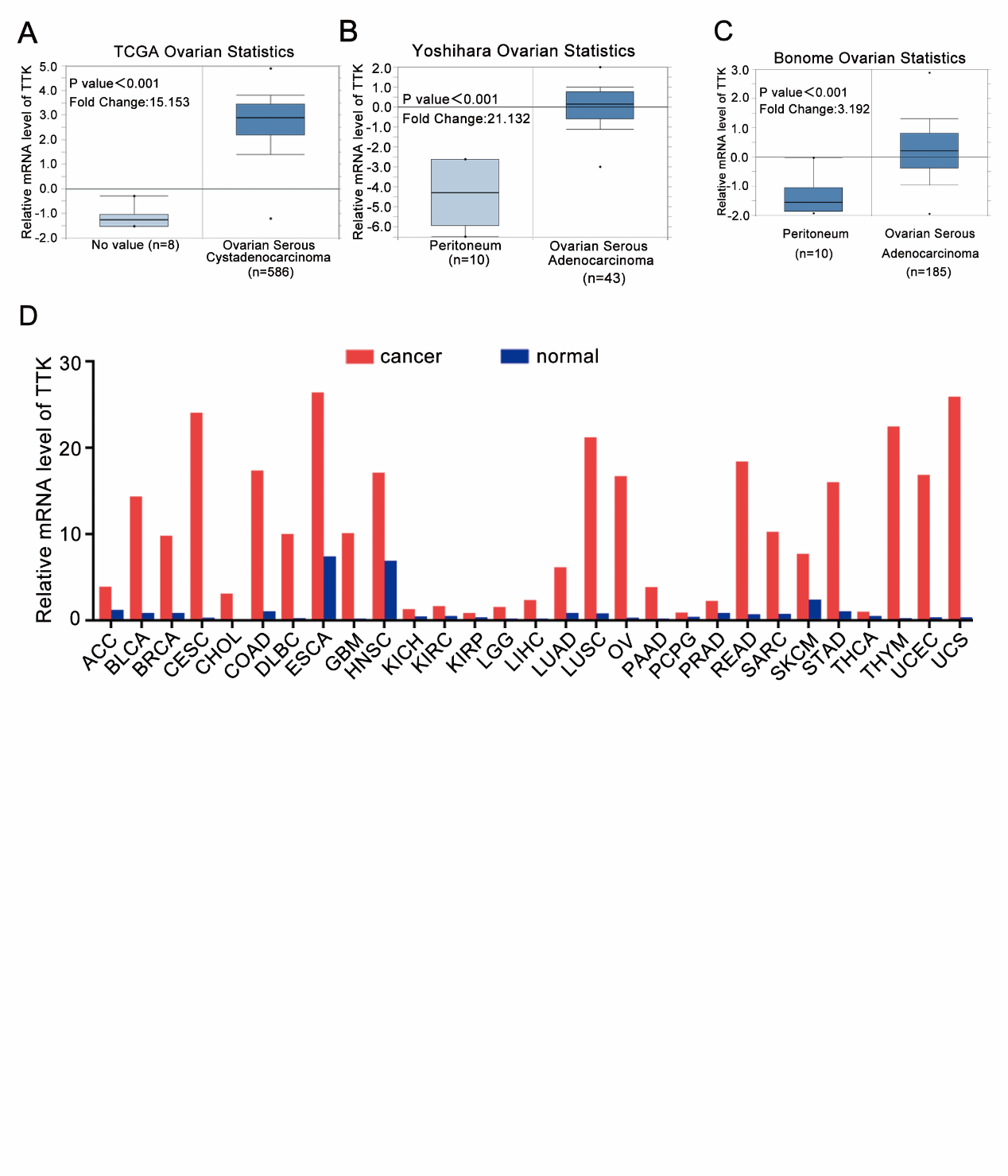


**Supplementary Figure S2**


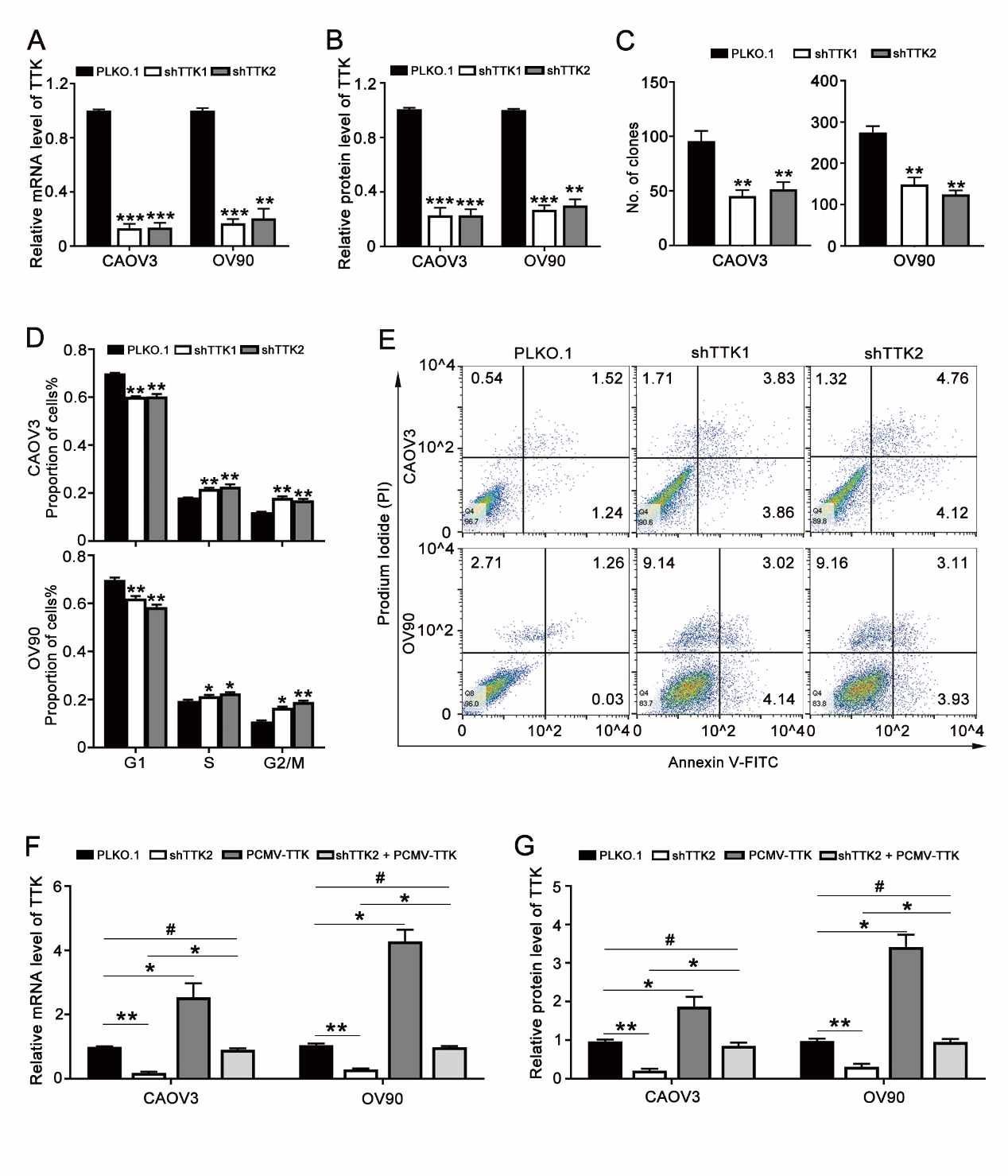


**Supplementary Figure S3**

**
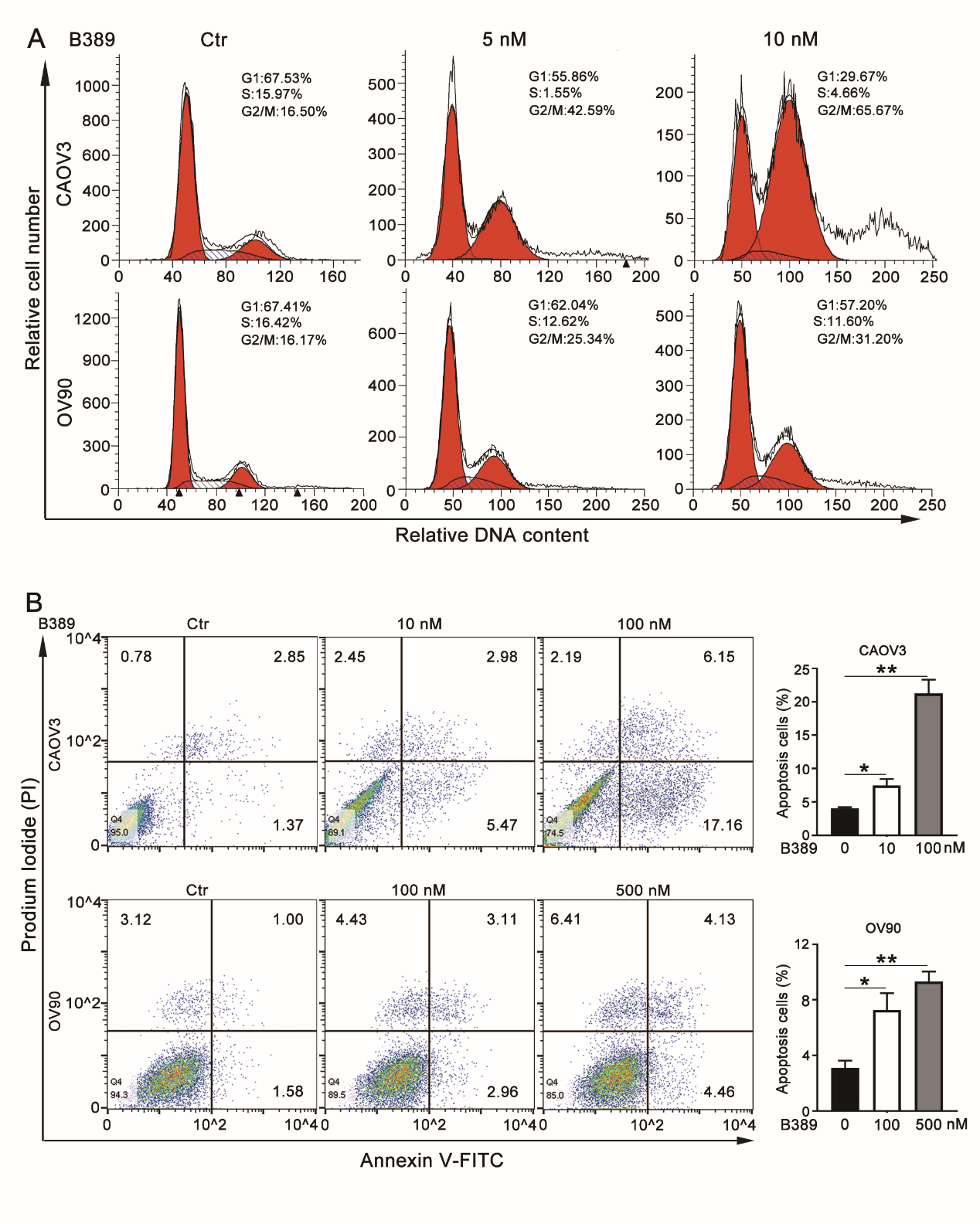
**

**Supplementary Figure S4**

**
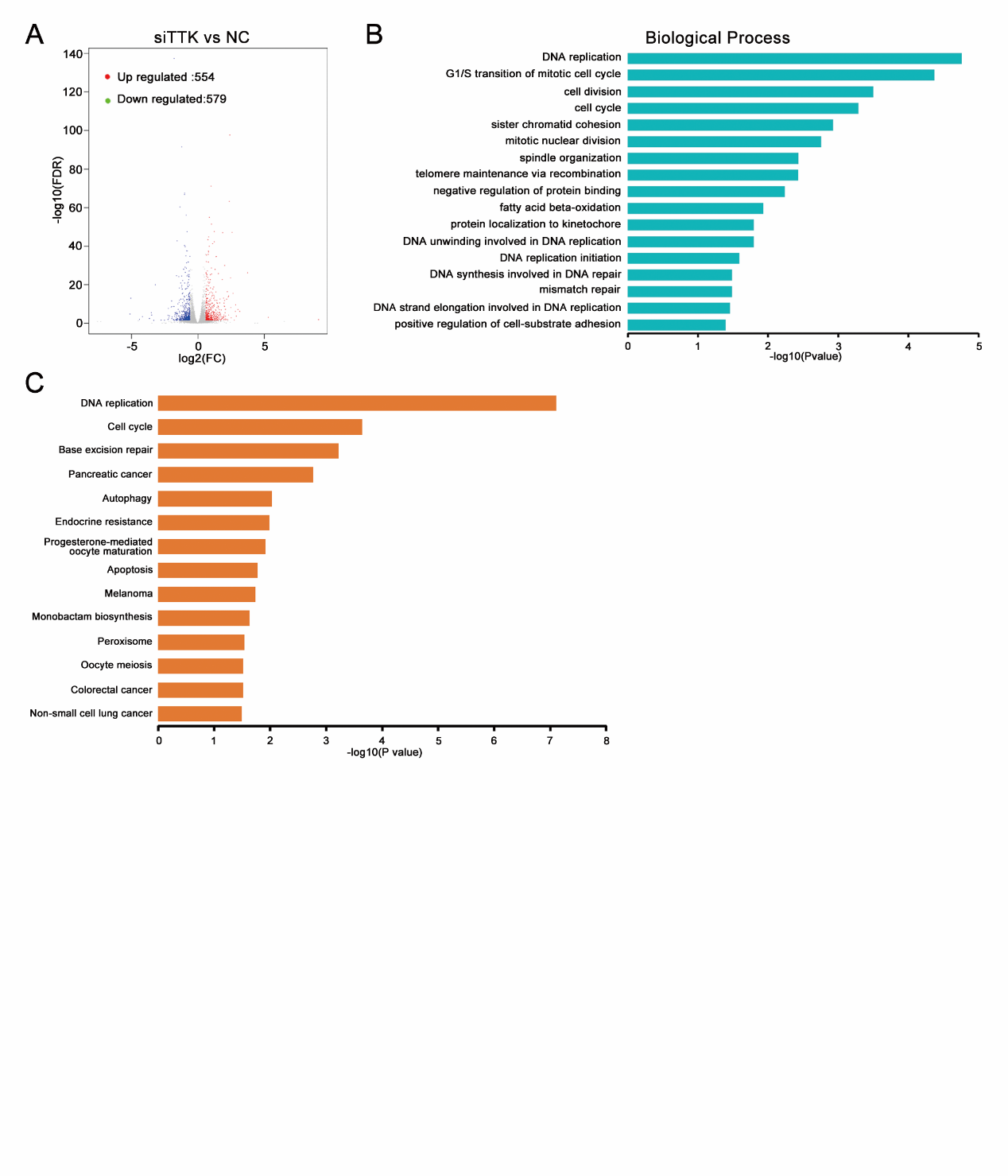
**

**Supplementary Figure S5**


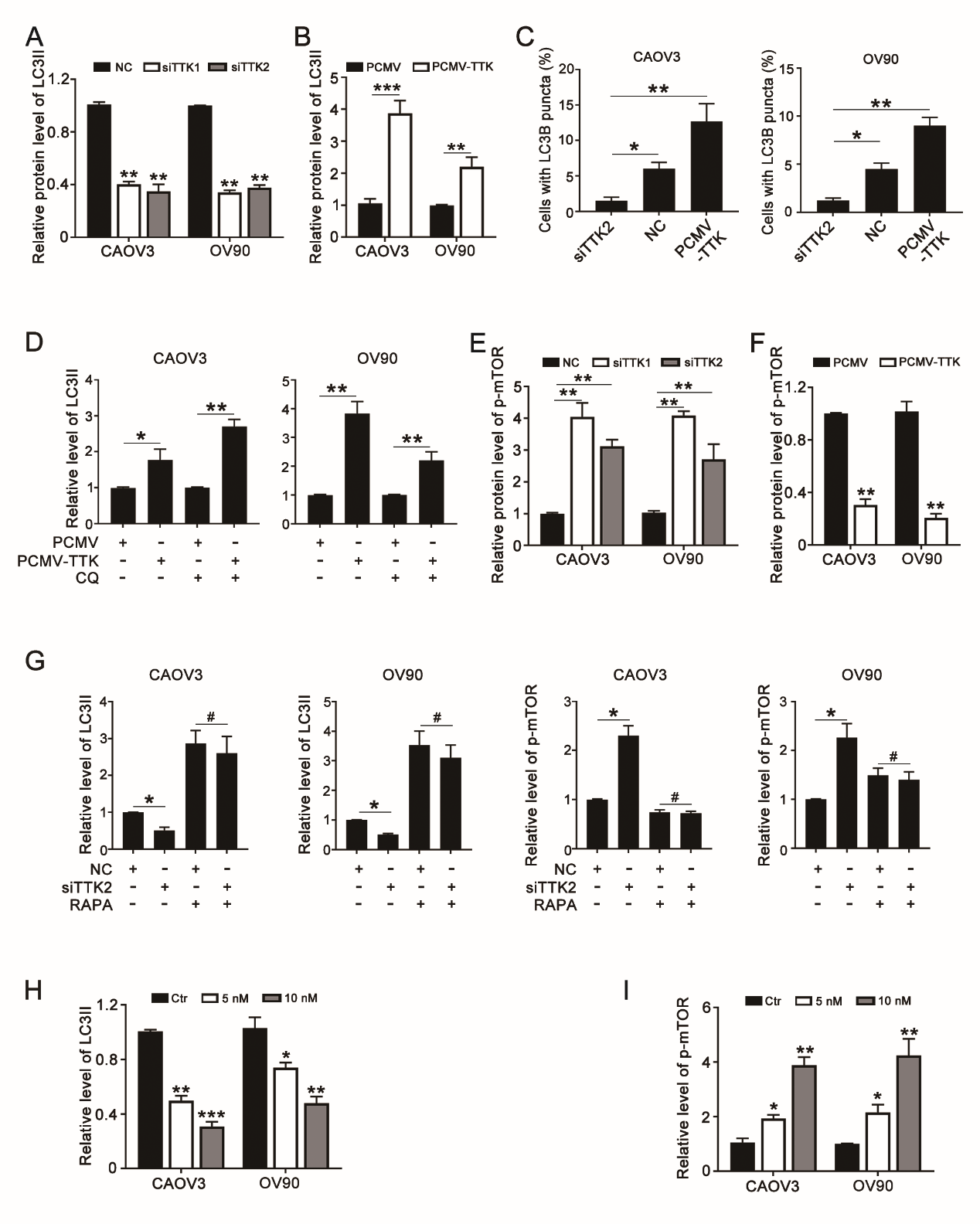


**Supplementary Table:**

**Supplementary Table S1. Differentially expressed genes (DEGs) in the cell cycle pathway.**

**Supplementary Table S1**. Differentially expressed genes (DEGs) in the cell cycle pathway.

| **Gene name** | **GSE14407 log2(FC)** | **GSE10971**  **log2(FC)** | **Inhibitors** | **Clinical trials of the inhibitors** | **Studies of ovarian cancer** |
| --- | --- | --- | --- | --- | --- |
| CDKN2A | 2.591223 | 3.847522 |  |  |  |
| BUB1B | 3.088582 | 3.152871 |  |  |  |
| TTK | 3.147243 | 2.704941 | YES | YES | NO |
| CDC7 | 1.651635 | 2.635227 | YES | YES | YES |
| CDC6 | 1.688575 | 1.060861 |  |  |  |
| CCNA2 | 2.049409 | 2.032875 |  |  |  |
| CDC20 | 2.440119 | 2.541065 | YES | NO |  |
| CCNB1 | 1.946837 | 2.120743 |  |  |  |
| ORC6 | 2.461326 | 1.372922 |  |  |  |
| CDC45 | 3.124138 | 1.602738 |  |  |  |
| ESPL1 | 2.782697 | 1.300503 |  |  |  |
| CCNE2 | 1.629966 | 2.014329 |  |  |  |
| PTTG1 | 2.816188 | 3.842652 |  |  |  |
| CCNE1 | 2.98276 | 3.853979 |  |  |  |
| CHEK1 | 1.354627 | 1.796903 | YES | YES | YES |
| E2F1 | 2.036195 | 2.647263 |  |  |  |
| CDK1 | 2.174597 | 2.683419 | YES | YES | YES |
| MCM4 | 2.006165 | 1.506987 |  |  |  |
| SFN | 3.205008 | 2.505683 |  |  |  |
| MCM6 | 1.245794 | 2.158245 |  |  |  |
| BUB1 | 1.785196 | 1.781471 | YES | NO |  |
| MCM2 | 1.744084 | 2.317601 |  |  |  |
