# Supplementary material for: TTK inhibition increases cisplatin sensitivity in high-grade serous ovarian carcinoma through the mTOR/autophagy pathway
Source: Cell Death Dis. 2021 Dec 7;12(12):1135. doi: 10.1038/s41419-021-04429-6 (PMC8651821; doi:10.1038/s41419-021-04429-6)
Supplement: Supplementary file 3 — author contribution [file 41419_2021_4429_MOESM3_ESM.pdf]

DECLARATION OF CONTRIBUTIONS TO ARTICLE

ADMC

Manuscript Number: DDIS-21-2154

Journal Name: *Cell Death & Disease* (the 'Journal')

Proposed Title of the Contribution: TTK inhibition increases cisplatin sensitivity through mTOR/autophagy pathway in ovarian cancer (the 'Contribution')

Author(s): Gonghua Qi, Hanlin Ma, Yingwei Li, Jiali Peng, Jingying Chen, Beihua Kong (the 'Authors')

For all *CDDis* articles, each person named as an author in the published version must be able to show he or she has contributed substantially to the article.

Authorship credit should be based on 1) substantial contributions to conception and design, acquisition of data, or analysis and interpretation of data; 2) drafting the article or revising it critically for important intellectual content; and 3) final approval of the version to be published. Authors should meet conditions 1, 2 and 3.

Any person who cannot be shown to have made a substantial contribution to the article cannot be listed as an author in the final version. The name of any person who is deemed to have made a minor contribution can, however, appear in the Acknowledgments section of the article.

Please complete the table below to indicate the contributions of all named authors to the manuscript.

Author Full Name:	Specification of Contribution to the Manuscript:
Gonghua Qi	Acquisition, analysis and interpretation of data; Drafting and final approval of the article
Hanlin Ma	Analysis and interpretation of data; Technical support; Revision and final approval of the manuscript
Yingwei Li	Interpretation of data; Technical support; Revision and final approval of the manuscript
Jiali Peng	Analysis and interpretation of data: Revision and final approval of the manuscript
Jingying Chen	Analysis and interpretation of data: Revision and final approval of the manuscript
Beihua Kong	Conception and design; Revision of the manuscript; Administrative, and material support; Final approval and Study supervision

扫描全能王 创建

Please complete the table below to indicate the contributions of all named authors to the figures.

Figure 1:

B.K generated the data and prepared panel A and B; Y.L generated the data and prepared panel C, G, H, I and J; G.Q generated other data; J.C labelled the image; H.M assembled the figure.

Figure 2:

G.Q generated the data; J.P labelled the image. H.M assembled the figure.

Figure 3:

G.Q generated the data and assembled the figure; J.C labelled the image.

Figure 4:

G.Q generated the data; J.C labelled the image. H.M assembled the figure.

Figure 5:

G.Q generated the data and assembled the figure; H.M labelled the image.

Figure 6:

G.Q generated the data; J.P and J.C labelled the image. H.M assembled the figure.

Signed for and on behalf of the Author(s):	Print Name:	Date:
Gonghua Qi; Hanlin Ma; Yingwei Li; Jiali Peng; Jingying Chen; Beihua Kong	Gonghua Qi, Hanlin Ma, Yingwei Li, Jiali Peng, Jingying Chen, Beihua Kong	2021/06/02

扫描全能王 创建
